# Long-term fate of long QT syndrome patients diagnosed in childhood and differential effects of beta-blockers

**DOI:** 10.3389/fcvm.2025.1732866

**Published:** 2026-01-12

**Authors:** Terézia Tavačová, Peter Kubuš, Petra Peldová, Markéta Pavlíková, Milan Macek, Alice Krebsová, Jan Janoušek

**Affiliations:** 1 Children’s Heart Centre, 2nd Faculty of Medicine, Charles University and Motol University Hospital, Prague, Czechia; 2 Department of Biology and Medical Genetics, 2nd Faculty of Medicine, Charles University and Motol University Hospital, Prague, Czechia; 3 Department of Probability and Mathematical Statistics, Faculty of Mathematics and Physics, Charles University, Prague, Czechia; 4 Cardiology Department, Institute for Clinical and Experimental Medicine, Prague, Czechia

**Keywords:** arrhythmias, child, long QT syndrome, prevention, sudden cardiac death

## Abstract

**Background and aims:**

Congenital long QT syndrome (LQTS) is associated with the risk of major arrhythmic events (MAEs), which can be modified by beta-blocker (BB) administration. We aimed to evaluate the effects of selective vs. non-selective BBs on the cumulative burden of MAEs by performing a retrospective analysis of a cohort of LQTS patients diagnosed during childhood.

**Methods:**

All pediatric patients (*N* = 179, female 54.7%) without structural heart disease diagnosed with LQTS (Schwartz score ≥3.5 points and/or presence of a pathogenic or likely pathogenic genetic variant) between 1985 and 2022, with a median presentation age of 10.8 and a median follow-up of 8.5 years, were included. Data were obtained from medical records, structured patient phone calls, and the National Death Registry.

**Results:**

Mean QTc and median Schwartz score were 501.20 ms and 4.0 points, respectively. Pathogenic or likely pathogenic variants were identified in 124 of 143 tested patients (86.7%). BBs were administered to 156 patients (87.2%). Eleven patients died (6.1%), yielding survival probabilities at 5/10/20 years of 97.7%/94.0%/90.2%. Freedom from MAEs was 93.9%/89.4%/79.3% at 5/10/20 years. MAE was independently predicted by early presentation [hazard ratio (HR): 6.92, *p* < 0.001], Schwartz score (HR: 1.87, *p* < 0.002), QTc (HR: 1.13, *p* < 0.001), and the presence of LQTS type 3 (HR: 7.91, *p* < 0.001). MAE burden was significantly lower in patients treated with non-selective vs. selective BBs, independent of other risk-determining variables (Poisson regression model *p* = 0.011).

**Conclusions:**

The 20-year survival probability of patients with LQTS diagnosed during childhood was 90.2% in the BB treatment era. Non-selective BBs were significantly more effective in decreasing the cumulative MAE burden.

## Introduction

Congenital long QT syndrome (LQTS) is an inherited arrhythmic disorder clinically characterized by delayed repolarization of cardiomyocytes, resulting in prolongation of the QT interval and a predisposing to life-threatening ventricular arrhythmias and sudden cardiac death (SCD) (1, 2). Pathogenic DNA variants in genes encoding ion channels involved in cardiomyocyte action potential formation are the primary culprits in the development of LQTS. The most commonly affected genes are the so-called significant LQTS genes *KCNQ1*, *KCNH2*, and *SCN5A* (3). Historically, the prevalence of the disease was estimated at 1:20,000 to 1:5,000, but subsequent data from Schwartz et al. suggested a prevalence of 1:2,000 (4).

LQTS remains a significant cause of sudden cardiac death in young individuals. Early diagnosis is essential to prevent malignant arrhythmias, enable accurate risk stratification, and identify relatives who may also carry pathogenic variants. Therapy with beta-blocking agents has been proven to significantly reduce the risk of life-threatening ventricular arrhythmias (5).

Over recent decades, several cohorts and registry studies have substantially advanced our understanding of the clinical risk stratification of congenital LQTS, revealing that therapeutic responses to different beta-blockers (BBs) vary across patients. Consistently identified predictors of major arrhythmic events include pronounced QTc prolongation, elevated Schwartz score, symptomatic presentation with syncope or aborted cardiac arrest, onset in infancy or early childhood, and LQT3 associated with *SCN5A* variants (1, 3, 6, 7).

More recent comparative work has demonstrated that BB therapy does not offer uniform protection in LQTS. Several multicenter analyses have reported a greater likelihood of breakthrough events among patients treated with cardioselective agents, particularly metoprolol, compared with older non-selective beta-blockers such as propranolol and nadolol. Propranolol has also been associated with more pronounced QTc shortening in higher-risk subgroups (6, 7). Meta-analyses and additional cohort data suggest that nadolol may be especially effective in LQT1 and LQT2, reinforcing the notion that BB efficacy is, at least in part, genotype-dependent. These observations have led many clinicians to preferentially prescribe non-selective BBs for pediatric and genotype-positive patients in recent years.

Despite these insights, direct comparisons of BB subtypes remain limited. Most prior investigations have focused on first arrhythmic events or composite outcomes, rather than evaluating the cumulative arrhythmic burden across different treatment phases. Our study addresses these gaps by examining both the time to first major arrhythmic event (MAE) and the overall MAE burden during periods of selective vs. non-selective BB therapy in a well-defined cohort of childhood-diagnosed LQTS.

The purpose of this study was therefore to describe the long-term clinical course of pediatric-onset LQTS, identify major risk factors for life-threatening arrhythmic events, and compare the effectiveness of selective vs. non-selective BB therapy.

## Materials and methods

### Study design

In the Czech Republic, pediatric patients (<19 years of age) with a suspected or definite LQTS diagnosis are referred to one of two national tertiary pediatric cardiovascular centers, according to regional referral practice. This retrospective observational study was performed at one of these tertiary referral centers—Children's Heart Centre, Motol University Hospital, Prague.

### Study population

A total of 271 patients were diagnosed with LQTS between July 1985 and January 2022. The following patients were subsequently excluded from the initial cohort: patients with any structural abnormality (*N* = 5), patients with incomplete cardiologic evaluation results (*N* = 11), patients subsequently reclassified to catecholaminergic polymorphic ventricular tachycardia (*N* = 3, pathogenic or likely pathogenic *RYR2* variant in all), and patients with a Schwartz score <3.5 (*N* = 67), unless a pathogenic or likely pathogenic variant was found by molecular-genetic evaluation. Patients with Andersen–Tawil syndrome (*N* = 6) were also excluded.

After these adjustments, the final study cohort comprised 179 remaining patients diagnosed with LQTS (176 Caucasians). Age at definite diagnosis was <19 years for all subjects. Diagnosis of LQTS for this study was established by achieving a Schwartz score ≥3.5 points (8–10) and/or by carrying a pathogenic or likely pathogenic variant associated with LQTS.

### Evaluation methods

All patients underwent a systematic diagnostic workup and follow-up protocol. The initial assessment included the individual's medical history, family history, clinical assessment, standard 12-lead electrocardiography, and transthoracic echocardiography to exclude any structural abnormalities in all our patients. A 12-lead ECG was recorded at a paper speed of 50 mm/s. RR and QT intervals were measured manually in leads II and V5, and the corrected QT interval (QTc) was calculated using Bazett's formula. The tangential method was used to assess the end of the T wave. If multiple examinations had been conducted, the longest QTc measured at rest and in the 4th min of recovery during the exercise stress test was selected.

An exercise test with QTc measured in the 4th min was available in 128 patients (71.5%). In the remaining 51 patients (28.5%), an initial exercise evaluation was not performed due to their young age, or unavailable results. These data were obtained to assess each patient's Schwartz score (9, 10).

### Follow-up and endpoint definitions

Follow-up data were retrieved from medical records and structured phone calls with patients/legal guardians. Cross-mapping with the National Death Registry enabled long-term survival analysis. The follow-up duration was defined as the interval between the first and last follow-up visits for patients still in the pediatric care, the date of the phone call for patients already transferred to adult care, or until death at any age. Information regarding medical history, symptoms, cardiovascular events, and genetic findings was retrieved from the institutional database and supplemented with information obtained through phone calls. The primary endpoint was death from any cause; the secondary endpoint was MAE, comprising sudden cardiac death, aborted cardiac arrest, documented ventricular fibrillation, sustained ventricular tachycardia, or appropriate implantable cardioverter-defibrillator (ICD) shock.

### Genetic testing

Phenotype-directed genetic testing (targeted mutation analysis) has been available at our center since 2014. Massively parallel sequencing was introduced in 2017 using a predefined cardiac gene panel, which was subsequently expanded to include clinical and whole-exome sequencing for more comprehensive analysis.

The respective gene variants were classified according to the guidelines of the American College of Medical Genetics and Genomics and the Association of Molecular Pathology (11). Pathogenic and likely pathogenic (P/LP) DNA variants were pooled in the analyses.

### Statistical analysis

Continuous variables were typically expressed as the median and interquartile range (IQR) or as the mean and standard deviation (SD), depending on the distribution pattern. Categorical variables were reported as absolute and relative frequencies (percentages). Kaplan–Meier analysis was used to assess survival probability, with statistical significance determined by the log-rank test. A Cox proportional hazards mixed regression model was used to estimate the contribution of clinical and genetic risk factors to the occurrence of first MAE and to compare the efficacy of selective vs. non-selective BBs. The model included the type of BB and one possible risk factor. A Poisson regression model was used to assess the effect of different BBs on MAE burden (12), accounting for multiple MAE occurrences and treatment periods with respective BB types. A *p-*value <0.05 was considered statistically significant.

### Ethics

The study was approved by the institutional review board and conducted in full compliance with the principles outlined in the Declaration of Helsinki. Informed consent was not required due to the retrospective nature of the study, its minimal impact on clinical patient management, and the fully anonymous presentation of data.

## Results

### Demography and clinical characteristics

**Figure 1:**
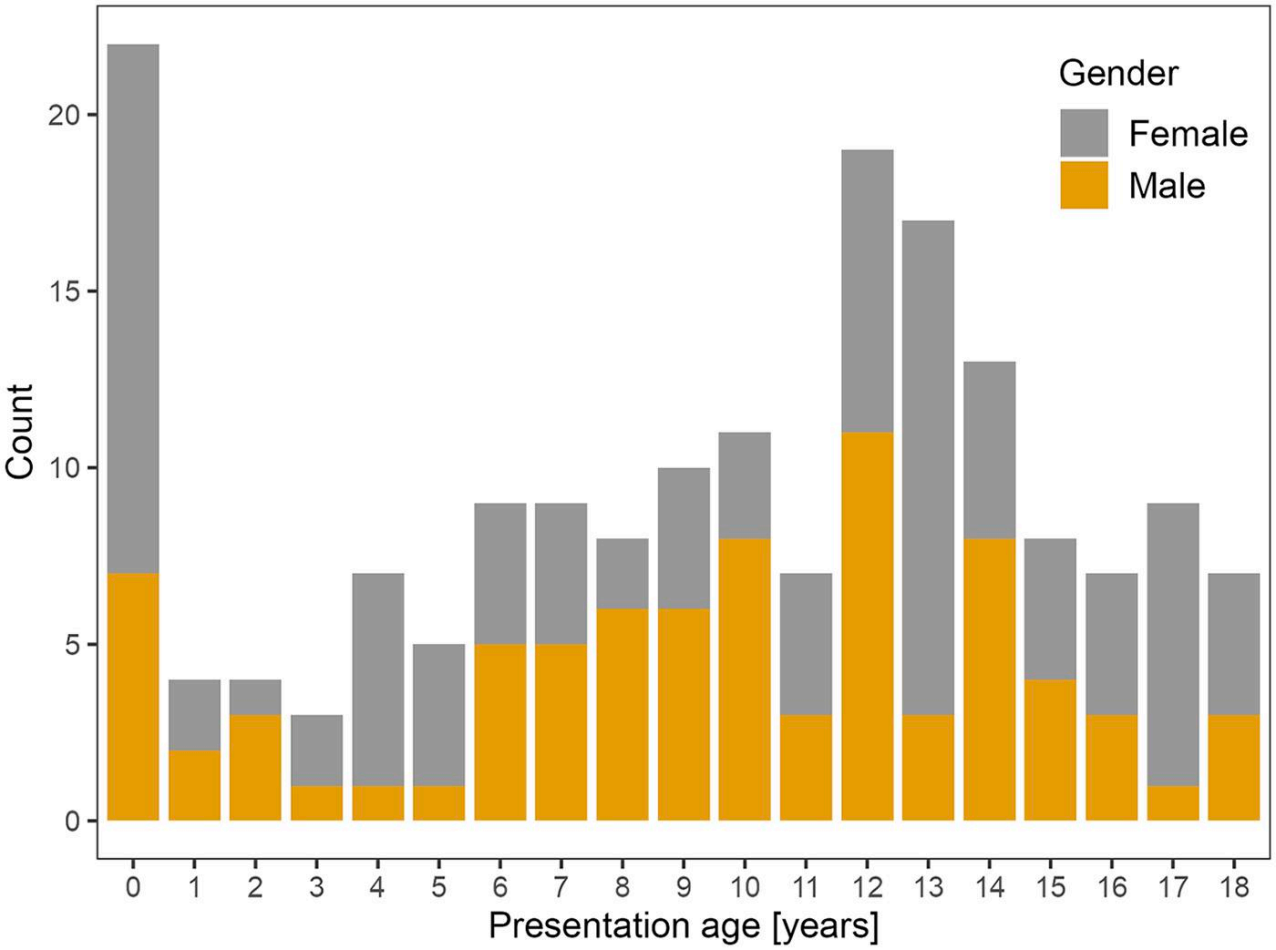
Distribution of the presentation age.

The data are summarized in Table 1. At presentation, the majority of the patients (*N* = 101, 56.4%) were asymptomatic; this group included 58 cases (32.4%) with a positive family history of LQTS, 19 patients (10.6%) referred following a positive preparticipation sport ECG screening of athletes, and 24 children (13.4%) with QTc prolongation found by coincidence, mostly during examination for heart murmur, hypertension, or chest pain caused by musculoskeletal etiology. Symptoms were present in 78 patients (43.6%): syncope with or without seizures in 43 cases (24.0%), sudden cardiac arrest with successful cardiopulmonary resuscitation in 23 patients (12.9%), bradycardia in six cases (3.4%), and palpitations also in six patients (3.4%). The distribution of age at presentation is illustrated in Figure 1.

**Table 1:** Demography and clinical characteristics.

Demography and clinical characteristics	Patients, *N* (%)
All patients, *N*	179
Gender, female/male, *N* (%)	98/81 (54.7/45.3)
Presentation age (years), median (IQR)	10.8 (6.0–13.8)
Presentation age, as category, *N* (%)	
Up to 30 days	11 (6.1)
31 days to <1 year	11 (6.1)
1–10 years	59 (33.0)
>10 years	98 (54.7)
QTc max (ms), mean (SD)	501.2 (53.5)
Schwartz score, median (IQR)	4.0 (4.0–5.0)
Symptoms at presentation, *N* (%)	78 (43.6)
Syncope	43 (24.0)
MAE	23 (12.8)
Asymptomatic (FH, sport PPE, incidental), *N* (%)	101 (56.4)
Molecular-genetic examination performed, *N* (%)	143 (79.9)
No LP/P variant found	19 (13.3)
LQTS1 (KCNQ1)	70 (39.1)
LQTS2 (KCNH2)	34 (19.0)
LQTS3 (SCN5A)	12 (6.7)
Other finding	8 (5.6)
MAE anytime during follow-up, *N* (%)	20 (11.2)
Death during follow-up, *N* (%)	11 (6.1)
Length of follow-up (years), median (IQR)	8.5 (5.3–16.9)
Beta-blockers administered anytime during follow-up, *N* (%)	156 (87.2)

FH, family history; IQR, interquartile range; MAE, major arrhythmic event; LP/P, likely pathogenic/pathogenic; PPE, preparticipation evaluation; SD, standard deviation.

### Molecular-genetic examination

**Figure 2:**
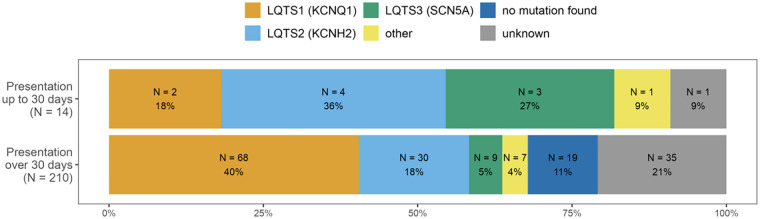
Genotype distribution in relation to the presentation age newborns vs. older children, LQTS 2 and 3 more prevalent in those presenting as newborns (*p* = 0.042).

Molecular-genetic testing was performed in 143 patients (79.9%). A pathogenic or likely pathogenic variant was identified in 124 of 143 patients, resulting in a diagnostic yield of 86.7%. Consistent with other studies (12, 13), the most commonly affected genes were *KCNQ1* in 70 out of 143 patients (49.0%), *KCNH2* in 34 patients (23.8%), and *SCN5A* in 12 patients (8.4%). Other variants (*KCNE1*, *SNTA1*, and *AKAP9*) were detected in eight children (5.6%). The proportion of *KCNH2* and *SCN5A* P/LP DNA variants was higher in patients presenting in the neonatal period (up to 30 days of life; *p* = 0.042) (Figure 2).

### Therapy

BBs were administered to 156 patients (87.2%). Metoprolol was used at any time in 92 patients (51.4%), nadolol in 74 (41.3%), atenolol in 45 (25.1%), propranolol in 26 patients (14.5%), metipranolol in 16 (8.9%), pindolol in 3 (1.7%), and acebutolol in 1 patient (0.6%). A significant number of patients (*N* = 52, 29.1%) were treated with more than one BB during their follow-up, reflecting the availability of drugs and changing treatment policies during the follow-up period, with a switch to non-selective BBs in recent periods. Consequently, the proportion of non-selective BB (metipranolol, nadolol, pindolol, and propranolol) increased from 16.7% to 58.5% during the periods 1985–2015 and 2016 onward (*p* < 0.001).

Other antiarrhythmic drugs used included mexiletine (*N* = 5, 2.8%) and flecainide (*N* = 2, 1.1%). Mexiletine was used as adjunctive therapy to BBs in patients all presenting in the neonatal period, four of them with pathogenic *SCN5A* variant and one carrying a pathogenic *KCNH2* variant (14, 15).

An antibradycardia pacemaker was implanted in 21 of 179 patients (11.7%). Thirteen patients received a pacemaker for bradycardia during the presentation period, most commonly for functional second-degree 2:1 AV block. The remaining eight underwent implantation later in adulthood, with the reason for implantation unknown. Seven of the 13 patients who presented with bradycardia had documented episodes of TdP and received VVI-ICD device therapy. Four neonates presenting with SCA/VT were supplied with a pacemaker device that lacked a defibrillating function due to size limitations.

A primary preventive ICD was used in eight high-risk patients (4.5%) with recurrent syncopes with clear arrhythmogenic etiology, QTc over 550 ms, or bradycardia requiring cardiac pacing for primary prevention. Another 19 patients (10.6%), including toddlers with bradycardia and documented TdP, received ICDs for secondary prevention. None of the patients underwent a left cardiac sympathetic denervation procedure.

### Survival

Eleven patients died from any cause (6.1%), yielding survival probabilities at 5/10/20 years of 97.7%/94.0%/90.2%, without a difference between female and male patients (*p* = 0.54) and not affected by the presentation age (neonatal, 1 month–10 years and >10 years of life, *p* = 0.75).

**Figure 3:**
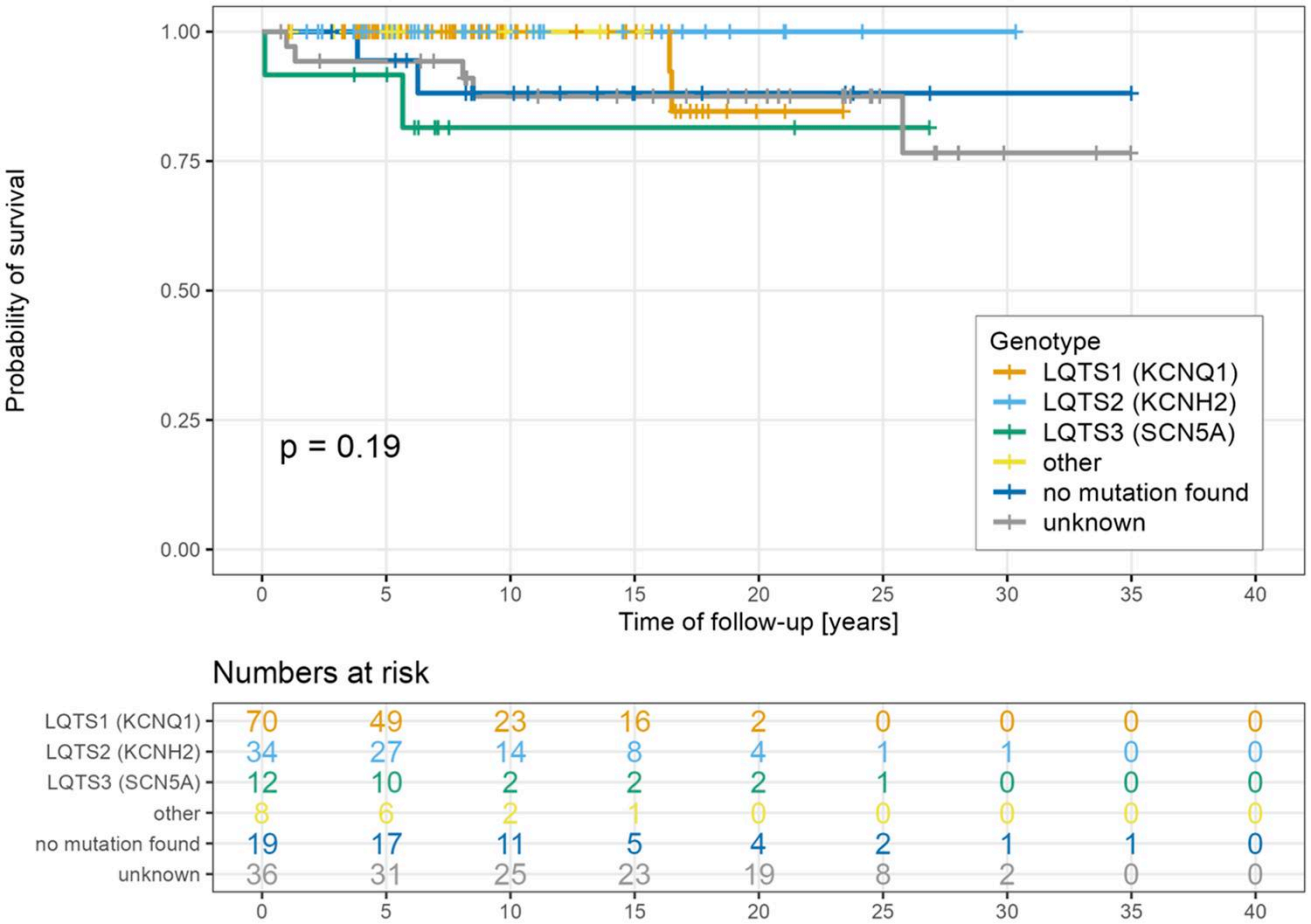
Survival probability according to genotype.

There was no statistically significant difference in overall survival probability among different types of LQTS (Figure 3).

### Major arrhythmic events

**Figure 4:**
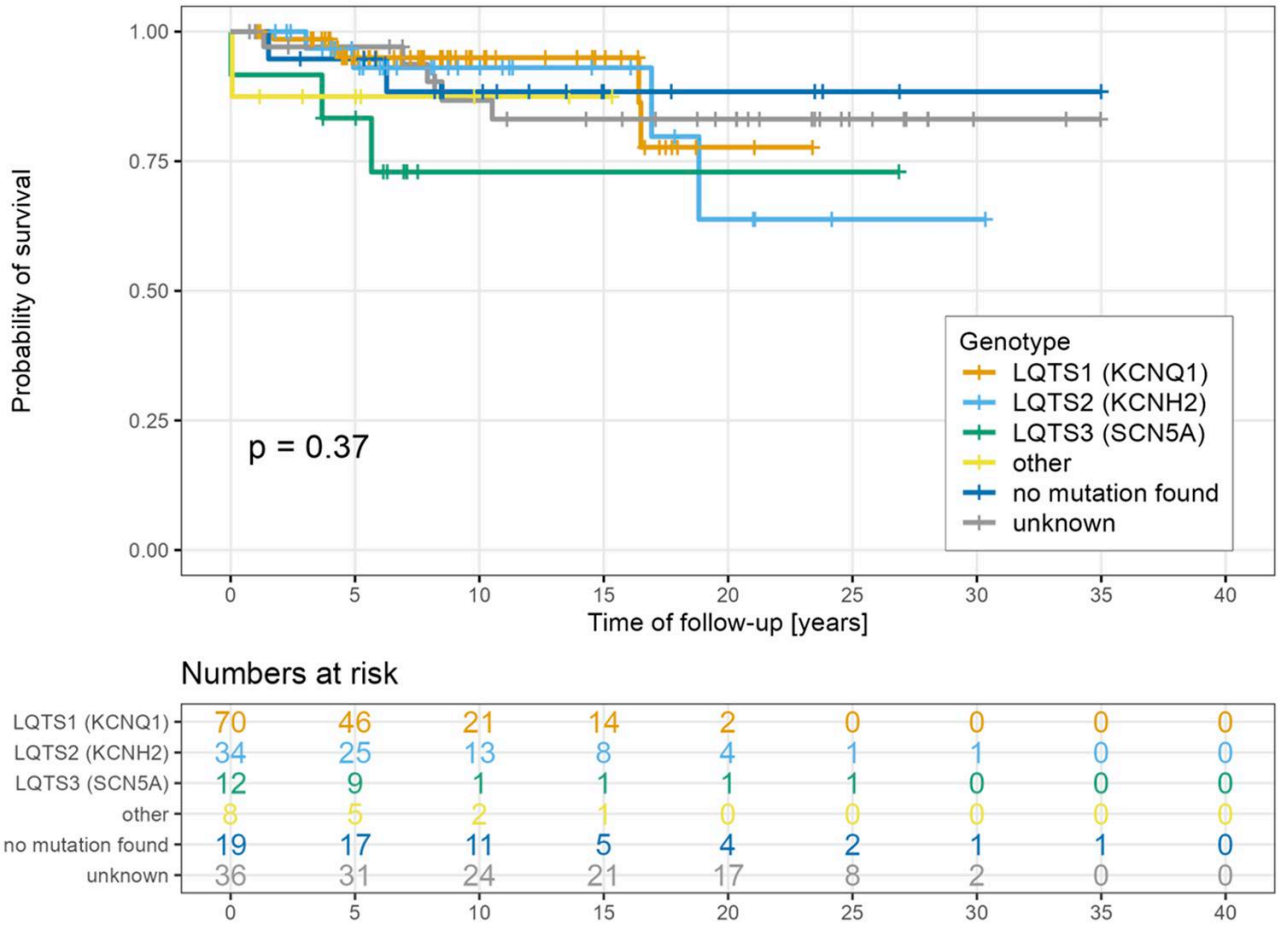
Freedom from MAE according to genotype.

**Figure 5:**
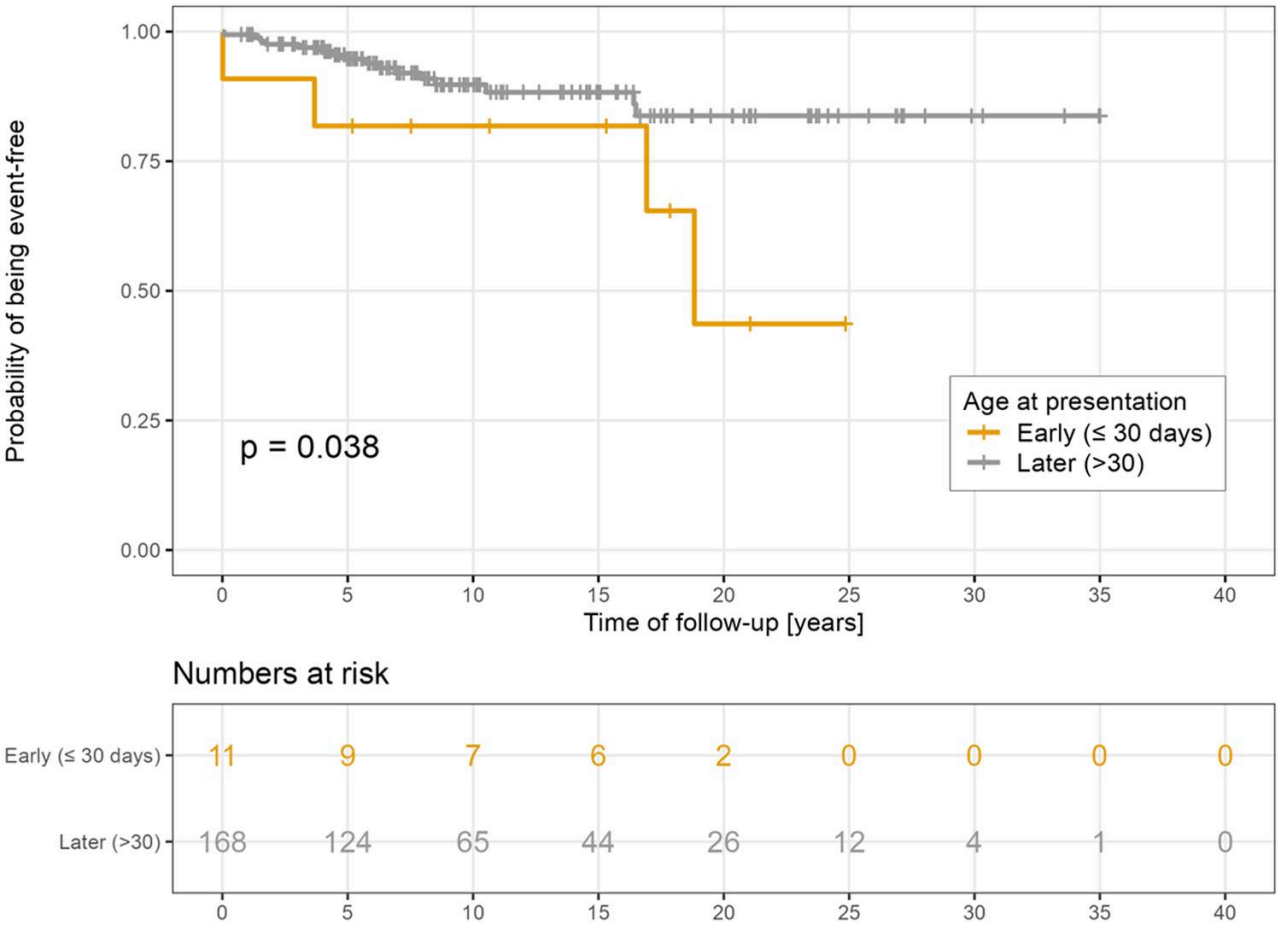
Freedom from MAE according to presentation age.

**Figure 6:**
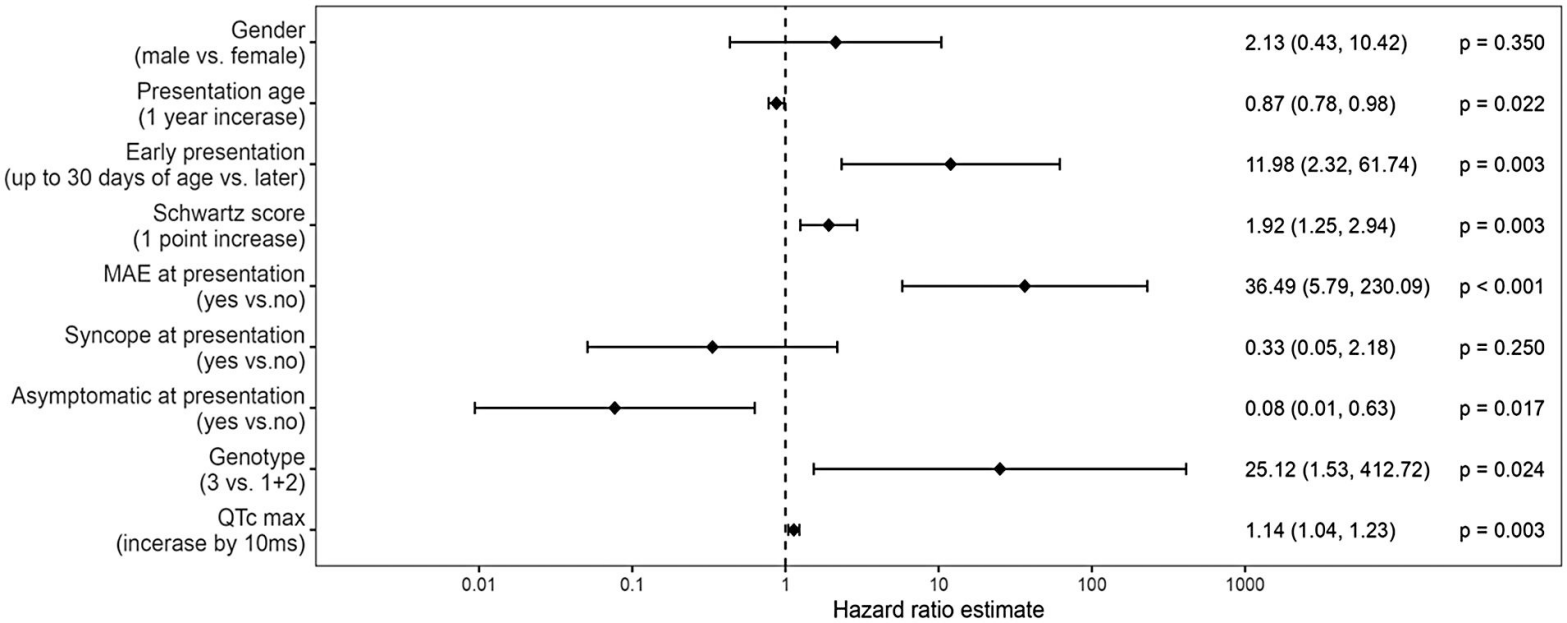
Hazard ratio estimates for prediction of MAE: demographic and clinical factors.

There was no significant difference in the probability of survival free from MAEs among different types of LQTS (Figure 4). However, patients presenting in the neonatal period had a significantly lower likelihood of freedom from MAEs as compared to the rest of the group (*p* = 0.038, Figure 5). Risk factors associated with the first occurrence of MAE under specific BB treatment included early presentation, higher Schwartz score, longer QTc, the presence of LQTS type 3, and the presence of ventricular arrhythmias at presentation (Figure 6).

### Effect of BB type on MAE burden

**Figure 7:**
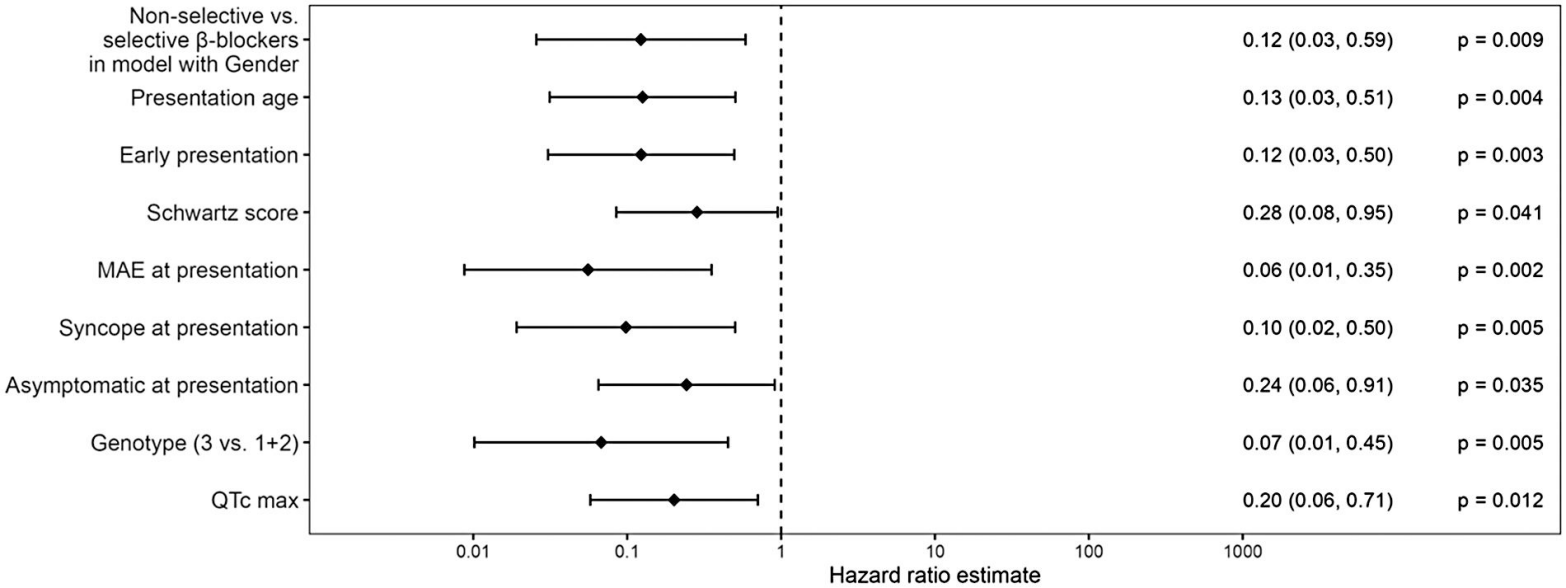
Hazard ratio estimates for prediction of MAE: non-selective vs. selective beta-blockers.

**Figure 8:**
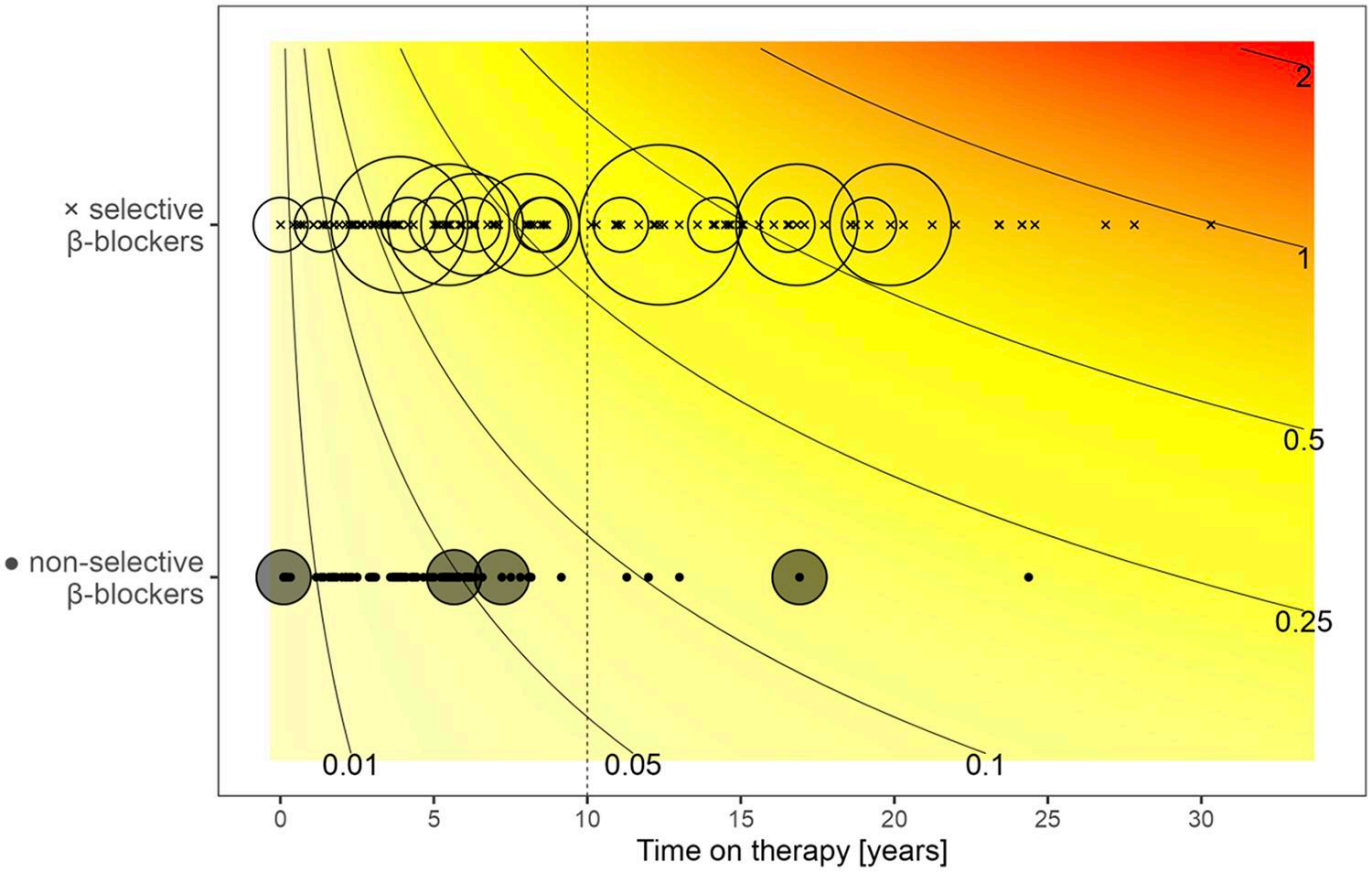
Beta-blocker type and cumulative MAE burden according to time on therapy. Analysis performed using Poisson regression model. Crosses (x) indicate a period of treatment with selective, whereas dots (·) indicate non-selective BBs. Circles around symbols represent the number of MAEs experienced during these periods. The size of the circle is proportional to the number of episodes. Isochrones indicate the estimated number of MAEs at respective treatment durations. During the 10-year treatment period, patients receiving non-selective BBs experienced 0.085 MAE episodes per patient as compared to 0.327 in patients on selective BB (12); the *p*-value for the difference between BB types is *p* = 0.011 for a mode assuming independence and *p* = 0.007 for a mixed model considering patient random effect.

**Figure 9:**
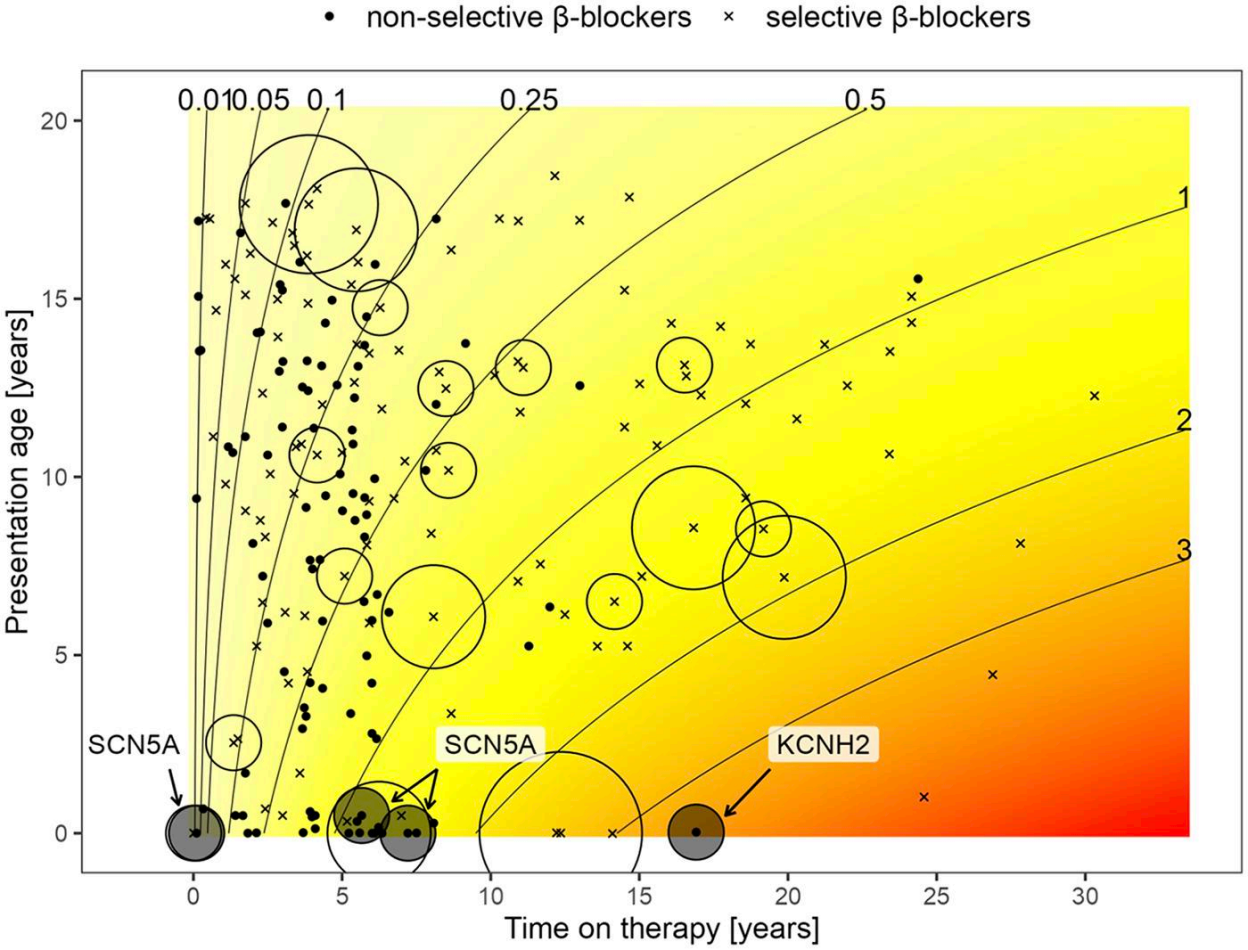
Cumulative MAE burden along with beta-blocker type, according to age at presentation and time on therapy. Analysis performed using a Poisson regression model. MAE episodes are most frequent in patients presenting early in life and occur, except few patients presenting as neonates, almost exclusively on selective beta-blockers. For a detailed explanation, see text and the legend for Figure 8.

Non-selective BBs (metipranolol, nadolol, pindolol, and propranolol) were more effective in reducing the risk of MAE as compared to selective BBs (acebutolol, atenolol, bisoprolol, and metoprolol), regardless of the presence of other risk factors (Figure 7). The cumulative MAE burden was also significantly lower during the non-selective BB treatment periods (*p* = 0.011, Figure 8). During a 10-year treatment period, patients receiving non-selective BBs experienced 0.085 MAE episodes per patient, compared with 0.327 episodes per patients in those treated with selective BBs. If analyzed according to the age at presentation (Figure 9), the only MAE occurring during non-selective BB therapy were in patients with highly malignant phenotypes presenting very early in life. Moreover, three of these patients carried an SCN5A mutation, for which the positive impact of BBs is limited, particularly in males (13, 16, 17).

## Discussion

Over the past few decades, considerable progress has been made regarding the diagnosis and management of LQTS. With appropriate treatment, an early and precise diagnosis of this condition can effectively prevent malignant cardiac events in these patients.

The first studies suggested a very malignant natural outcome in untreated patients, with mortality rates of 71% in LQTS patients (8). BB therapy has since been established as an effective way of preventing SCD (5, 13, 16, 18). The survival probability of the reported cohort treated with BBs ranged between 97.7% and 90.2% over 5–20 years of follow-up. Given the cohort character, these figures represent a real-world long-term survival scenario in the era of BB treatment in clinical practice. The studied group appears representative of the general LQTS population, with 43.6% of the patients symptomatic at presentation (12.9% with aborted cardiac arrest) (19, 20). Consistent with published studies (21), our cohort's most commonly affected genes were (in descending order) *KCNQ1*, *KCNH2*, and *SCN5A*. The yield of genetic testing (86.7%) matched the reported data in patients with a well-marked phenotype (22).

In line with available studies (3, 23, 27), risk factors for MAEs included early presentation, VT or SCA at presentation, LQTS type 3, higher Schwartz score, and longer QTc.

Previous studies have shown that patients with LQTS type 1 generally have a more favorable prognosis, with higher overall and MAE-free survival rates, compared with individuals with LQTS types 2 and 3 (3). In our cohort, however, no statistically significant differences in overall survival probability were observed across the three major genotypes (Figure 3). This finding may, in part, reflect the effectiveness of contemporary genotype-specific management strategies, but it is also likely influenced by the limited sample sizes within each LQTS subtype.

In contrast to previous studies, we evaluated the protective BB effect in terms of the first MAE episode and analyzed its efficacy in reducing the cumulative MAE burden using a Poisson regression model. Such analysis allowed us to examine various treatment periods in the same patient who switched from one BB type to another, thereby better reflecting true long-term treatment efficacy. This statistical approach confirmed a clear benefit of non-selective BBs. The only patients experiencing MAEs on non-selective BBs were those with highly malignant phenotypes presenting very early in life, with most carrying an *SCN5A* mutation. To the best of our knowledge, this is the first study using a Poisson regression model in LQTS patients.

Our finding that treatment with non-selective BBs was significantly more protective, regardless of other risk-determining variables, and was associated with a lower cumulative MAE burden extends prior comparative studies (6, 7). Chockalingam et al. observed a higher recurrence of cardiac events under metoprolol compared with propranolol and nadolol, with greater QTc shortening reported with propranolol. Abu-Zeitone and colleagues likewise compared different BBs within an international registry and found genotype-dependent differences in efficacy, with nadolol performing exceptionally well in the LQT1 and LQT2 subgroups. Our results are consistent with these reports and make an additional novel contribution: The effect persists when MAE burden (recurrent events over time) is modeled using a Poisson regression model, and the benefit is observed in the pediatric-diagnosed cohort followed into adolescence/adulthood.

Several pharmacodynamic properties may explain why non-selective β-blockers (e.g., nadolol, propranolol) appear more effective than cardioselective β1 agents (e.g., atenolol, metoprolol) in reducing MAEs in LQTS. Nadolol and propranolol block both β1 and β2 receptors. In specific LQTS triggers (sympathetic response, exercise), β2 blockade in the myocardium and vascular system may provide additional stabilization of repolarization and reduce heterogeneity of ventricular refractoriness beyond β1 blockade alone (24). Several selective agents or older drugs (e.g., acebutolol, pindolol) also exhibit intrinsic sympathomimetic activity (ISA), which can produce partial agonist effects and may thus be less suppressive of adrenergic-mediated arrhythmogenesis. Most non-selective agents used in reported LQTS cohorts (nadolol, propranolol) lack ISA (25). Moreover, propranolol has been shown to inhibit voltage-gated sodium channels in cardiac tissue at therapeutic concentrations, producing a direct antiarrhythmic membrane-stabilizing effect that is independent of β-receptor antagonism. This mechanism may be particularly relevant for arrhythmias arising from sodium current abnormalities (including some SCN5A phenotypes) or for suppressing triggered activity (26). In addition, nadolol has a long half-life and relatively stable plasma levels with once-daily dosing, which can provide more consistent adrenergic blockade over 24 h compared with shorter-acting agents (e.g., metoprolol), particularly if dosing adherence is imperfect (7). Together, these properties explain the superior effectiveness of non-selective BBs observed in multiple cohorts and in the present analysis.

While BBs are highly effective in LQT1 (IKs channel defects), their effectiveness is less pronounced in LQT3 (*SCN5A* gain-of-function). The few MAEs observed on non-selective agents in our cohort clustered in early-presenting, highly malignant *SCN5A* phenotypes, consistent with prior reports that *SCN5A* carriers remain at relatively higher residual risk despite therapy (26).

In our cohort, the Poisson model estimated ∼0.085 MAEs per patient over 10 years on non-selective BB vs. ∼0.327 MAEs per patient on selective BB (*p* = 0.011), a clinically meaningful difference that aligns directionally with those prior reports and with meta-analytic syntheses. Notably, since a subset of patients switched between BB types during follow-up, our within-patient modeling of treatment periods reinforces the argument that the observed difference is driven by drug characteristics rather than solely by baseline patient selection.

According to the results of the mentioned studies, many centers now use non-selective BBs as the preferred first-line treatment for high-risk LQTS patients, particularly in children and in patients with LQT1 or LQT2. If nadolol cannot be used, propranolol is a reasonable alternative, provided adequate dosing is ensured, to achieve a 24-h beta-blockade. For patients with *SCN5A*-related LQT3, BBs offer less protection overall, so additional genotype-specific treatments (such as mexiletine) or device therapy may be necessary (7).

BBs are the mainstay of therapy for LQTS and are recommended for nearly all affected children; however, specific scenarios require avoidance, dose adjustment, or careful monitoring. Neonates and infants with very low resting heart rates are at risk of pauses that may trigger TdP, which can be prevented with antibradycardia pacing. This was implemented in 13 patients in our cohort. No sinus node dysfunction was observed.

Exercise intolerance was managed by adjusting the dose, guided by repeated exercise testing, to maintain adequate β-blockade. Bronchospasm, a known adverse effect of non-selective BBs, was addressed through dose modification or, in some cases, switching to selective BBs. These complications were infrequent in the pediatric population.

Direct randomized comparisons of specific BB therapy remain lacking. Available data are observational and subject to confounding by indication, era (drug availability and prescribing patterns have changed over time), and dosing differences. Our retrospective design is therefore susceptible to such biases despite statistical adjustment and within-patient treatment period modeling. Nevertheless, the consistency of results across different centers, combined with our own Poisson model analysis of cumulative burden, makes a compelling case for preferential use of non-selective BBs in LQTS patients.

## Limitations

One of the significant limitations of the study is its retrospective character. Six-monthly to annual follow-ups were available for all patients during the pediatric period (i.e., less than 19 years of age). However, to obtain the follow-up data for the adult patients, we had to contact them via structured phone calls, where some information may have been missed. Moreover, in some patients, only scanned ECGs of poor quality were available, which may have led to inaccuracies in the QTc measurements.

The survival analysis in this group of patients reflects the course of the disease in patients with a definite or highly probable diagnosis of LQTS (carrying a likely pathogenic/pathogenic variant in one of the genes associated with LQTS or a Schwartz score ≥3.5 points) who were subjected to BB treatment in the vast majority. Other patients with milder/questionable phenotypes and a negative genotype were excluded, which could potentially interfere with the results.

Survival may have been influenced by complications associated with cardiac implantable electronic devices used in a subset of the cohort. As only data on all-cause mortality have been available without specific reasons for death, the influence of the respective factors could not be analyzed.

## Conclusion

Patients with LQTS diagnosed during childhood and treated with BBs in the vast majority had a long-term survival probability of about 90%. In our study group, early presentation, QTc duration, higher Schwartz score, symptoms at presentation, and LQTS type 3 were major predictors of MAEs. Genetic testing demonstrated a high diagnostic yield. Over time, BB therapy has shifted to non-selective BB. Non-selective BB therapy was more effective than selective BB in significantly decreasing the MAE burden, regardless of other risk-determining variables.

## Data Availability

The original contributions presented in the study are included in the article/Supplementary Material, further inquiries can be directed to the corresponding author.

## References

[1] Schwartz PJ Crotti L Insolia R. Long-QT syndrome: from genetics to management. Circ Arrhythm Electrophysiol. (2012) 5:868–77. 10.1161/CIRCEP.111.962019 22895603 PMC3461497

[2] Moss AJ. Long QT syndrome. JAMA. (2003) 289:2041–4. 10.1001/jama.289.16.2041 12709446

[3] Priori SG Schwartz PJ Napolitano C Bloise R Ronchetti E Grillo M Risk stratification in the long-QT syndrome. N Engl J Med. (2003) 348(19):1866–74. 10.1056/NEJMoa022147 12736279

[4] Schwartz PJ Stramba-Badiale M Crotti L Pedrazzini M Besana A Bosi G Prevalence of the congenital long-QT syndrome. Circulation. (2009) 120:1761–7. 10.1161/CIRCULATIONAHA.109.863209 19841298 PMC2784143

[5] Moss AJ Zareba W Hall WJ Schwartz PJ Crampton RS Benhorin J Effectiveness and limitations of beta-blocker therapy in congenital long-QT syndrome. Circulation. (2000) 101(6):616–23. 10.1161/01.cir.101.6.616 10673253

[6] Chockalingam P Crotti L Girardengo G Johnson JN Harris KM van der Heijden JF Not all beta-blockers are equal in managing long QT syndrome types 1 and 2: higher recurrence of events under metoprolol. J Am Coll Cardiol. (2012) 60(20):2092–9. 10.1016/j.jacc.2012.07.046 23083782 PMC3515779

[7] Abu-Zeitone A Peterson DR Polonsky B McNitt S Moss AJ. Efficacy of different beta-blockers in the treatment of long QT syndrome. J Am Coll Cardiol. (2014) 64(13):1352–8. 10.1016/j.jacc.2014.05.068 25257637

[8] Schwartz PJ. Idiopathic long QT syndrome: progress and questions. Am Heart J. (1985) 109(2):399–411. 10.1016/0002-8703(85)90626-x 3966369

[9] Schwartz PJ Moss AJ Vincent GM Crampton RS. Diagnostic criteria for the long QT syndrome: an update. Circulation. (1993) 88(2):782–4. 10.1161/01.cir.88.2.782 8339437

[10] Schwartz PJ Crotti L. QTc behavior during exercise and genetic testing for the long-QT syndrome. Circulation. (2011) 124(20):2181–4. 10.1161/CIRCULATIONAHA.111.062182 22083145

[11] Richards S Aziz N Bale S Bick D Das S Gastier-Foster J Standards and guidelines for interpreting sequence variants: a joint consensus recommendation of the American College of Medical Genetics and Genomics and the Association for Molecular Pathology. Genet Med. (2015) 17(5):405–24. 10.1038/gim.2015.30 25741868 PMC4544753

[12] Hayat MJ Higgins M. Understanding Poisson regression. J Nurs Educ. (2014) 53(4):207–15. 10.3928/01484834-20140325-04 24654593

[13] Tester DJ Ackerman MJ. Genetics of long QT syndrome. Methodist Debakey Cardiovasc J. (2014) 10:29–33. 10.14797/mdcj-10-1-29 24932360 PMC4051331

[14] Mazzanti A Maragna R Faragli A Monteforte N Bloise R Memmi M Gene-specific therapy with mexiletine reduces arrhythmic events in patients with long QT syndrome type 3. J Am Coll Cardiol. (2016) 67(9):1053–8. 10.1016/j.jacc.2015.12.033 26940925 PMC4773513

[15] Bos JM Crotti L Rohatgi RK Castelletti S Dagradi F Schwartz PJ Mexiletine shortens the QT interval in patients with potassium channel-mediated type 2 long QT syndrome. Circ Arrhythm Electrophysiol. (2019) 12(5):e007280. 10.1161/CIRCEP.118.007280 31006312

[16] Wilde AA Moss AJ Kaufman ES Shimizu W Peterson DR Benhorin J Clinical aspects of type 3 long-QT syndrome: an international multicenter study. Circulation. (2016) 134(12):872–82. 10.1161/CIRCULATIONAHA.116.021823 27566755 PMC5030177

[17] Hermida A Gourraud JB Denjoy I Fressart V Kyndt F Maltret A Type 3 long QT syndrome: is the effectiveness of treatment with beta-blockers population-specific? Heart Rhythm. (2024) 21(3):313–20. 10.1016/j.hrthm.2023.11.007 37956775

[18] Priori SG Wilde AA Horie M Cho Y Behr ER Berul C HRS/EHRA/APHRS expert consensus statement on the diagnosis and management of patients with inherited primary arrhythmia syndromes: document endorsed by HRS, EHRA, and APHRS in May 2013 and by ACCF, AHA, PACES, and AEPC in June 2013. Heart Rhythm. (2013) 10:1932–63. 10.1016/j.hrthm.2013.05.014 24011539

[19] Horigome H Nagashima M Sumitomo N Yoshinaga M Ushinohama H Iwamoto M Clinical characteristics and genetic background of congenital long-QT syndrome diagnosed in fetal, neonatal, and infantile life: a nationwide questionnaire survey in Japan. Circ Arrhythm Electrophysiol. (2010) 3(1):10–7. (Erratum in: Circ Arrhythm). 10.1161/CIRCEP.109.882159 19996378

[20] Rohatgi RK Sugrue A Bos JM Cannon BC Asirvatham SJ Moir C Contemporary outcomes in patients with long QT syndrome. J Am Coll Cardiol. (2017) 70(4):453–62. 10.1016/j.jacc.2017.05.046 28728690

[21] Adler A Novelli V Amin AS Abiusi E Care M Nannenberg EA An international, multicentered, evidence-based reappraisal of genes reported to cause congenital long QT syndrome. Circulation. (2020) 141(6):418–28. 10.1161/CIRCULATIONAHA.119.043132 31983240 PMC7017940

[22] Tester DJ Will ML Haglund CM Ackerman MJ. Effect of clinical phenotype on yield of long QT syndrome genetic testing. J Am Coll Cardiol. (2006) 47(4):764–8. 10.1016/j.jacc.2005.09.056 16487842

[23] Goldenberg I Moss AJ Peterson DR McNitt S Zareba W Andrews ML Risk factors for aborted cardiac arrest and sudden cardiac death in children with the congenital long-QT syndrome. Circulation. (2008) 117(17):2184–91. 10.1161/CIRCULATIONAHA.107.701243 18427136 PMC3944375

[24] Han L Liu F Li Q Qing T Zhai Z Xia Z The efficacy of beta-blockers in patients with long QT syndrome 1–3 according to Individuals’ gender, age, and QTc intervals: a network meta-analysis. Front Pharmacol. (2020) 11:579525. 10.3389/fphar.2020.579525 33381033 PMC7768040

[25] Frishman WH. Clinical significance of beta 1 selectivity and intrinsic sympathomimetic activity in a beta-adrenergic blocking drug. Am J Cardiol. (1987) 59(13):33F–7F. 10.1016/0002-9149(87)90039-7 2883876

[26] Wang DW Mistry AM Kahlig KM Kearney JA Xiang J George AL Jr. Propranolol blocks cardiac and neuronal voltage-gated sodium channels. Front Pharmacol. (2010) 1:144. 10.3389/fphar.2010.00144 21833183 PMC3153018

[27] Hobbs JB Peterson DR Moss AJ McNitt S Zareba W Goldenberg I Risk of aborted cardiac arrest or sudden cardiac death during adolescence in the long-QT syndrome. JAMA. (2006) 296(10):1249–54. 10.1001/jama.296.10.1249 16968849

