# Supplementary material for: Long-term fate of long QT syndrome patients diagnosed in childhood and differential effects of beta-blockers
Source: Front Cardiovasc Med. 2026 Jan 12;12:1732866. doi: 10.3389/fcvm.2025.1732866 (PMC12833094; doi:10.3389/fcvm.2025.1732866)
Supplement: Supplementary file 1 [file Image1.pdf]

Long-term fate of patients with LQTS diagnosed in childhood and differential effect of beta-blockers (BB)

179 paediatric LQTS patients

Male/Female	81 /98
Age at presentation	mean 10.8 yrs
QTc max	mean 501.2 ms
Schwartz score	median 4.0
Symptomatic	78 (43.6 %)
LQTS 1/2/3	116 (64.8 %)

MAE – major arrhythmic event (suden cardiac arrest/death/ICD shock therapy)

Event-free survival probability according to presentation age

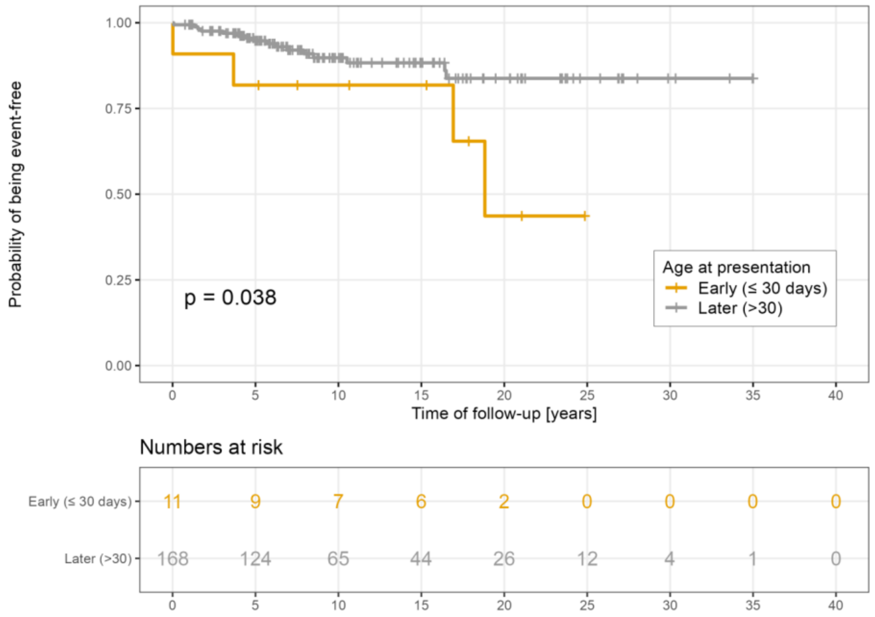

BB type and cumulative burden of MAE

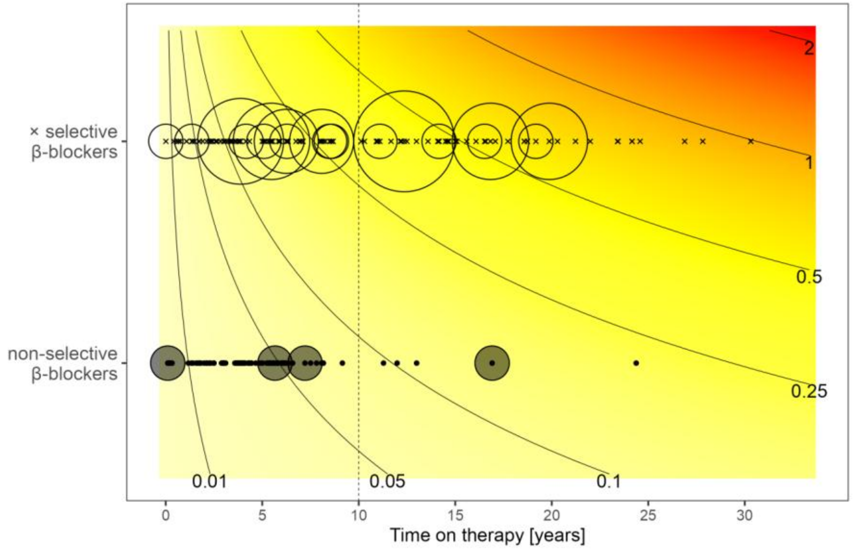

Estimated rate of MAE in 10 years: 0.327 for selective and 0.085 for nonselective BBs

Non-selective betablockers are preferable in paediatric LQTS patients regardless of genotype and clinical profile
